# Anti-Aging and Antioxidant Potential of *Paullinia cupana* var. *sorbilis*: Findings in *Caenorhabditis elegans* Indicate a New Utilization for Roasted Seeds of Guarana

**DOI:** 10.3390/medicines4030061

**Published:** 2017-08-15

**Authors:** Herbenya Peixoto, Mariana Roxo, Teresa Röhrig, Elke Richling, Xiaojuan Wang, Michael Wink

**Affiliations:** 1 Institute of Pharmacy and Molecular Biotechnology, Heidelberg University, INF 364, D-69120 Heidelberg, Germany; hspeixoto1@gmail.com (H.P.); marianaroxocorreia@gmail.com (M.R.); wxjsz@hotmail.com (X.W.); 2 Department of Food Chemistry and Toxicology, Molecular Nutrition, University of Kaiserslautern, Erwin-Schroedinger-Strasse 52, D-67663 Kaiserslautern, Germany; roehrig@chemie.uni-kl.de (T.R.); richling@chemie.uni-kl.de (E.R.)

**Keywords:** *C. elegans*, lifespan, oxidative stress, anti-aging, *Paullinia cupana*, guarana

## Abstract

**Background:** Roasted seeds of Amazonian guarana (*Paullinia cupana* var. *sorbilis;* Sapindaceae) are popular in South America due to their stimulant activity on the central nervous system (CNS). Rich in purine alkaloids, markedly caffeine, the seeds are extensively used in the Brazilian beverage industry for the preparation of soft drinks and as additives in energy drinks. **Methods:** To investigate the putative anti-aging and antioxidant activity of guarana, we used the model organism *Caenorhabditis elegans*. Chemical analyses were performed using high-performance liquid chromatography (HPLC) and electrospray ionization-mass spectrometry (ESI-MS/MS). **Results:** When tested in the model system *Caenorhabditis elegans*, the water extract from roasted guarana seeds enhanced resistance against oxidative stress, extended lifespan and attenuated aging markers such as muscle function decline and polyQ40 aggregation. **Conclusions:** In the current study, we demonstrate that guarana extracts can work as a powerful antioxidant in vivo; moreover, guarana extracts exhibit anti-aging properties. Our results suggest that the biological activities of guarana go beyond the extensively reported CNS stimulation.

## 1. Introduction

Plants that produce purine alkaloids like caffeine, theophylline and theobromine are widely used due to their stimulant activity on the central nervous system (CNS). Among the most popular plants we can list coffee (*Coffea arabica*), tea (*Camellia sinensis*), mate (*Ilex paraguariensis*) and cocoa (*Theobroma cacao*) which are mainly consumed as beverages and teas. They are also found included in sweets, bakery and desserts all over the world. The stimulant effects of purine alkaloids are related to their ability to inhibit cAMP phosphodiesterases and adenosine receptors promoting, among others, an increase in mental alertness, physical performance and reduction of fatigue [1,2]. Due to their pharmacological properties, isolated purine alkaloids are active ingredients of several medical preparations, as well as nutraceuticals and energy drinks of high popularity among those who search for natural products to achieve health and wellness goals. However, indiscriminate consumption is not free of adverse effects [3].

Guarana [*Paullinia cupana* var. *sorbilis* (Mart.) Kunth; Sapindaceae], widely used in the Brazilian beverage industry, is reported to have the highest concentration of caffeine (more than 6% in the seeds) observed in nature so far. The seeds are famous in Brazil for their stimulant effects. Popularly, roasted seed powder is used in preparations to enhance memory and to attenuate mental and physical stress [4].

Guarana is native to the Amazonia forest and was domesticated by indigenous people of the ethnic group Sateré Mawé about 500 years ago. In the wild, it grows as a woody vine that can reach 10 m in length, but when domesticated it grows as a bush reaching approximately two to three meters in height. It bears a characteristic red colored fruit that when ripe partially opens, exhibiting up to three black seeds covered at their lower half with a thick white aril, resembling an eye [5].

Espinola et al. [6] assessed the effects of guarana on the physical and cognitive performance of rats. The anti-fatigue effect was demonstrated in a forced swimming test; surprisingly, the group treated with pure caffeine or ginseng did not show a significant difference from the untreated control. Additionally, the authors demonstrated that guarana is readily able to reverse the amnesic effect of scopolamine. Otobone et al. [7] tested the effects of guarana seed extract on rats submitted to a Morris water maze test and reported significantly lower latency to escape among treated animals in comparison with untreated ones. Otobone et al. [8] demonstrated the antidepressant effect of guarana in rats and Rangel et al. [9] provided evidence for anxiolytic effects. Besides the effects on the CNS, guarana can attenuate markers of metabolic syndrome, like waist circumference and cholesterol levels; this effect was demonstrated among seniors self-referred as guarana drinkers. The authors additionally reported a significant association between lower levels of advanced oxidative protein products and guarana consumption [10]. Ruchel et al. [11] reported the ability of guarana to reduce total cholesterol and LDL-cholesterol to basal levels in hypercholesterolemic rats and highlighted the association between high guarana intake and reduction of the inflammatory process in those hypercholesterolemic rats. 

Other activities frequently credited to guarana include analgesic, aphrodisiac, astringent, anorectic, anti-inflammatory, anti-platelet, cardiotonic, bronchorelaxant, gastrostimulant, immunostimulant, thermogenic and diuretic properties [12]. Carvalho et al. [13] reported antiproliferative activity of guaraná crude extract in HL-60 cells.

Considering the high amount of caffeine in guarana, it sounds reasonable to credit its biological effects to the purine alkaloids. However, guarana also contains significant amounts of phenolics, especially tannins like catechin, epicatechin and their polymers and proanthocyanidins, which might also contribute to its properties that go beyond the CNS stimulant effect [14,15,16].

In the current study, we have investigated a water extract and an alkaloid extract from roasted seeds of guarana (*P. cupana* var. *sorbilis*) with regard to its putative antioxidant and anti-aging properties. To determine antioxidant activity, the guarana extract was analyzed in vitro by DPPH method, a standard antioxidant assay. The in vivo antioxidant activity was investigated using the nematode *C. elegans* that is a powerful system in this context [17,18,19]. Furthermore, we evaluated a potential protective effect of the guarana extract against induced oxidative stress, its influence on the cellular accumulation of reactive oxygen species (ROS) and in the expression of important stress response genes like *hsp-16.2* and *sod-3,* which is regulated by DAF-16/FOXO transcription factor. To explore a potential anti-aging activity, we investigated the influence of guarana extract on the lifespan of *C. elegans*, age-related muscle function decline and formation of polyQ40 plaques, which are considered as sensitive and relevant aging markers.

## 2. Material and Methods

### 2.1. Plant Material and Extraction

Guarana extract (GE) was prepared from the powder of roasted seeds purchased from a local trader in Manaus-AM (Brazil). The powder was weighted and exhaustively extracted with distilled water (5 × 1 L) at room temperature during an overall extraction period of 5 d. Using a rotary evaporator, the extract was reduced to approximately ¼ of the initial volume at low pressure, 40 °C, and subsequently frozen at −80 °C. The frozen extract was lyophilized yielding a fine dried powder. The alkaloid extract (AlkE) was obtained through standard alkaline dichloromethane extraction.

### 2.2. Chemical Characterization of the Extract

#### 2.2.1. Characterization of Alkaloids

The alkaloid content (caffeine, theobromine and theophylline) of GE was determined by high-performance liquid chromatography-ultraviolet/visible spectroscopy (HPLC-UV/VIS) using an Agilent 1200 series HPLC system (Agilent, Santa Clara, CA, USA), provided with a column Synergi 4 μm Polar-RP 80 Å, 250 × 4.6 mm (Phenomenex, Torrance, CA, USA). Solvent system A—0.1% formic acid, solvent B—acetonitrile. The gradient profile was: 2–12% B over 5 min, 12–30% B over 15 min, 30–90% B over 3 min, isocratic 90% B for 8 min, 90–2% over 1 min, isocratic 2% B for 3 min. The flow rate was 0.8 mL/min and the column temperature was kept at 40 °C, the injection volume was 20 μL. The sample concentration was 1 mg/mL diluted in water. The UV-detection wavelength was 270 nm. The measurements were carried out in duplicate. The standard addition calibration method was used to determine the concentration of the analyzed compounds. Pure reference alkaloids were available and used for peak matching.

#### 2.2.2. Characterization of Catechins (ESI-MS/MS)

Catechins and procyanidins in the guarana extract were identified with an Agilent 1100 HPLC system (Santa Clara, CA, USA) equipped with a degasser, quaternary pump, autosampler, column compartment, and VW-detector coupled to a PE Sciex API 2000 triple quad mass spectrometer (Sciex, Framingham, MA, USA). HPLC conditions were partly adapted from (Bonoli, et al., 2003). HPLC conditions: column Luna 5 μm C18 100 Å, 250 × 4.6 mm (Phenomenex, Torrance, CA, USA); column temperature: 20 °C; solvent system: A-H_2_O/methanol/formic acid: 74.7/25/0.3 (*v*/*v*/*v*), B—acetonitrile/formic acid: 99.7/0.3 (*v*/*v*); gradient profile: isocratic 0% B for 8 min, 100% B over 24 min, isocratic 100% B for 6 min, 0% B over 4 min, isocratic 0% B for 6 min; flow rate: 0.5 mL/min; injection volume: 20 μL; sample concentration: 1 mg/mL in H_2_O. ESI-MS/MS conditions: positive ion mode; ion spray voltage: 5500 V; temperature 450 °C; declustering potential: 61 V; focusing potential: 370 V; entrance potential: 12 V; collision cell entrance potential: 14 V [20].

#### 2.2.3. Total Phenol Content

The Folin-Ciocalteu method was adapted to 96-well microplate format. Briefly, 100 μL of Folin-Ciocalteu reagent (Merck, Darmstadt, Germany) was added to 20 μL of sample; 5 min later, 80 μL of sodium carbonate (7.5% solution) were added. The reaction ran in the dark at room temperature for 2 h. The absorbance was measured at 750 nm using a microplate reader (Tecan Group Ltd., Männedorf, Switzerland). All measurements were carried out in triplicate and at least three times. The total phenol content is expressed as gallic acid equivalents (GAE/mg of sample).

### 2.3. Antioxidant Activity in Vitro (DPPH Assay)

A method described by Blois [21] was adapted to a 96-well microplate format. Briefly, 100 μL of 200 μM DPPH (2,2-diphenyl-1-picrylhydrazyl; Sigma-Aldrich GmbH, Steinheim, Germany) was added to 100 μL of sample. The reaction ran in the dark at room temperature (25 °C) for 30 min and the absorbance was measured at 517 nm using a microplate reader. The scavenging activity was calculated according the following equation:

DPPH scavenging effect (%) = [(A0 – A1)/A0] × 100

where A0 means the absorbance of the control reaction and A1 is the absorbance in the presence of AE. All measurements were performed in triplicate. The EC_50_ value was estimated by sigmoid non-linear regression and is presented in μg/mL.

### 2.4. Caenorhabditis Elegans: Strains and Culture

The strains N2 (wt), CF1038 [daf-16(mu86)I], GR1307 [daf-16(mgDf50)-I], CF1553 {muls84 [pAD76(sod-3::GFP)]}, TJ375 (gpIs1[hsp-16-2::GFP]), BA17 [(fem-1(hc17)IV] and AM141 [rmIs133 (unc-54p::Q40::YFP)] were purchased from the Caenorhabditis Genetics Center (CGC, University of Minnesota, Minneapolis, MN, USA). Worms were cultured on solid nematode growth media (NGM) plates, fed on living *Escherichia coli* OP50 and maintained at 20 °C in a temperature-controlled incubator [22]. Prior to the assays, the eggs were isolated to obtain age synchronized cultures and kept in M9 buffer for hatching as described by Strange et al. [23]. For all assays, after 24 h at 20 °C the larvae (L1 stage) were transferred to liquid S-medium inoculated with living *E. coli* OP50 (O.D._600_ = 1.0) and treated according to the correspondent group.

### 2.5. Antioxidant Activity in Vivo

#### 2.5.1. Survival Assay under Oxidative Stress 

Age synchronized worms (strains N2, CF1038 and GR1307), grown in S-medium at L1 larval stage, were sorted into groups of 75 worms and treated with GE or AlkE for 48 h, except the control. Then, the groups were exposed to 80 μM of the pro-oxidant juglone (5-hydroxy-1,4-naphthoquinone; Sigma-Aldrich GmbH, Steinheim, Germany) and the number of dead and live worms was counted for 24 h after juglone treatment. The worms were considered dead when they did not respond to a gentle touch with a platinum wire. The assay was carried out in triplicate and results are presented as mean ± SEM, compared by one-way analysis of variance (ANOVA) followed by Bonferroni (post-hoc).

#### 2.5.2. Intracellular ROS Accumulation 

Age synchronized N2 worms (L1 stage, grown in S-medium) were sorted into groups and treated with GE or AlkE for 48 h, except the control group. Afterwards, the worms were washed with M9 buffer and incubated with 1 mL 50 μM CM-H2DCFDA solution (1 h at 20 °C) (6-carboxy-2′,7′-dichlorodihydrofluorescein diacetate; Fluka Chemie GmbH, Buchs, Switzerland), washed again with M9 and mounted on a glass slide, and a drop of 10 mM sodium azide (AppliChem GmbH, Darmstadt, Germany) was added to paralyze the nematodes. Live images were captured from at least 30 worms per group, using a fluorescence microscope (Keyence Deutschland GmbH, Neu-Isenburg, Germany; λEx 480/20 nm; λEm 510/38 nm). The relative fluorescence of the whole body was determined densitometrically using ImageJ software version 1.48 (National Institutes of Health, Bethesda, MD, USA). The results are presented as mean fluorescence intensity (mean ± SEM) and were compared by one-way ANOVA followed by Bonferroni (post-hoc). The assay was repeated three times.

### 2.6. Expression of Stress Response Genes

#### 2.6.1. Quantification of hsp-16.2::GFP Expression

Age synchronized worms (TJ375, L4 stage, grown in S-medium) which carry a GFP reporter fused with *hsp-16.2* were sorted into groups and treated with GE or AlkE for 48 h, except the control group. Subsequently, 20 μM juglone was added to the medium and 24 h later the worms were submitted to fluorescence microcopy as described before.

#### 2.6.2. Quantification of Sod-3::GFP Expression

Age synchronized worms (CF1553, L1 stage, grown in S media) which carry a GFP reporter fused with *sod*-3, were treated with GE or AlkE for 48 h, except the control group, and submitted to fluorescence microscopy as described above.

### 2.7. Aging Markers

#### 2.7.1. Longevity Assay

Age synchronized worms (BA17, grown in S-medium, at day 1 of adulthood) were sorted and treated with GE, except the control group. Live worms were transferred every second day to fresh medium and counted during the transfer. The worms exhibiting internally hatched progeny or extruded gonads were scored as censored worms and excluded from the assay. Dead worms, those that did not respond to a gentle touch with the platinum wire, were scored and removed from the assay. The results are presented as percentage of survival and the statistical significance was determined by Log-rank (Mantel-Cox) tests followed by Gehan-Breslow-Wilcoxon Test.

#### 2.7.2. Pharyngeal Pumping Rate

Age synchronized worms (N2, day 1 of adulthood, grown in S-medium) were sorted and placed on NGM agar plates seeded with *E. coli* OP50. The bacterial lawn was supplemented with GE in the treated groups. The adults were transferred daily to separate them from their progeny; after day 5 of adulthood, the transfer was carried out one day before the analyses of the pumping activity, which were taken on day 5, 8 and 10 of adulthood. To score the pumping rate, each worm was observed for 1 min when crawling on the bacterial lawn using a stereoscope. Each group had a minimum of 10 worms. The results are presented as pumps/min (mean ± SEM) and were compared by two-way ANOVA followed by Bonferroni (post-hoc).

#### 2.7.3. Quantification of PolyQ40::YFP Aggregates

Age synchronized worms (transgenic strain AM141), cultured at 20 °C in S-medium inoculated with living *E. coli* OP50, at L1 stage, were separated in groups and treated for 48 h with GE, except the control group. Then, the worms were mounted onto a glass slide with a drop of 10 mM sodium azide for paralysis and submitted to fluorescence microscopy (λEx 480/20 nm; λEm 510/38 nm). The results are presented as number of PolyQ40::YFP aggregates (mean ± SEM) and were compared by one-way ANOVA followed by Bonferroni (post-hoc).

### 2.8. Toxicity Assay: C. Elegans Brood Size

Age synchronized worms (N2, L4 stage, grown in S-medium) were sorted and placed individually on NGM agar plates seeded with *E. coli* OP50. The bacterial lawn was supplemented with GE or AlkE in the treatment groups. From day 1 of adulthood, the worms were transferred to fresh NGM plates supplemented according to their groups and the eggs at the former NGM plates were scored. The procedure was repeated for 5 days. The mean brood size was compared by two-way ANOVA followed by Bonferroni (post-hoc).

## 3. Results and Discussion

### 3.1. Chemical Characterization of Guarana Extract

Using HPLC-UV/Vis, three compounds were identified in guarana alkaloid extracts, namely caffeine, theobromine and theophylline. The quantification showed a high concentration of caffeine (102.8 mg/g), followed by theophylline (2.3 mg/g) and theobromine (1.0 mg/g). ESI-MS/MS analysis detected the presence of catechin and epicatechin (Table 1). Our results thus agree with the published chemical composition of guarana [14,15,24] and highlight the high concentration of caffeine as a hallmark of the species.

The Folin-Ciocalteu method demonstrates a high phenolic content (1250 GAE/g of sample) for the roasted seed extract. Total phenolic content correlates with the antioxidant activity observed in vitro and in vivo.

### 3.2. In Vitro Antioxidant Activity of Guarana

Guarana extract was evaluated regarding its antioxidant activity in vitro through DPPH assay (Table 2) and exhibited moderate activity when compared with standard dietary antioxidants such as vitamin C and epigallocatechin gallate (EGCG). The alkaloid extract (AlkE) however, exhibited no antioxidant activity (Table 2).

### 3.3. In Vivo Antioxidant Activity of Guarana

**Figure 1:**
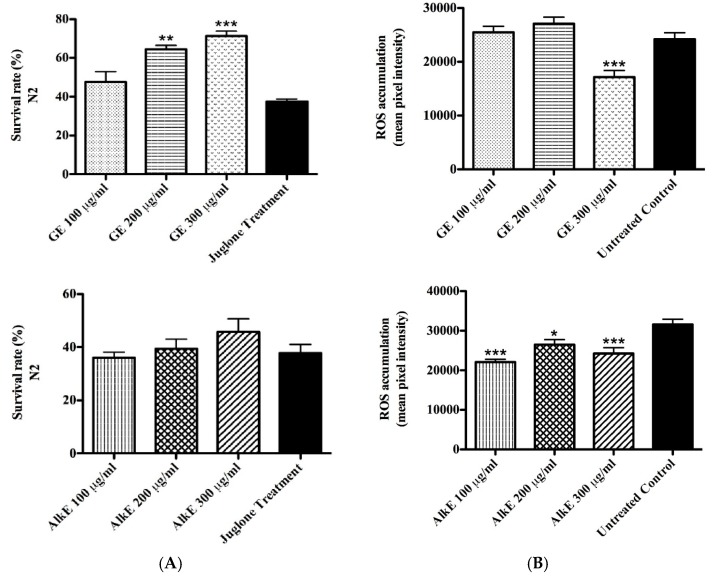
Effect of GE and AlkE on stress resistance of wild-type worms. (**A**) Survival rate of N2 (wt) worms under juglone-induced oxidative stress. Survival rate of N2 worms was significantly enhanced in the groups treated with 200 and 300 μg/mL of GE; no significant difference was observed among worms treated with AlkE and untreated worms. (**B**) Intracellular reactive oxygen species (ROS) accumulation in N2 (wt) worms after treatment with GE. ROS accumulation was significantly decreased by GE and AlkE treatment compared to the untreated control. The results are presented as mean ± SEM from three independent experiments. * *p* < 0.05, ** *p* < 0.01 *** *p* < 0.0001 compared to the untreated control by one-way analysis of variance (ANOVA) followed by Bonferroni’s method (post-hoc).

The antioxidant effects of GE were investigated in vivo in *C. elegans*. Worms were treated with a lethal dose of the toxic pro-oxidant juglone and analyzed 24 h later regarding the score of dead and live individuals. Worms pre-treated with GE exhibited a significantly higher survival rate as compared to the untreated control. At the highest tested concentration of 300 μg/mL GE, the survival rate was up to 71%, while in untreated control worms it was about 37% (adjusted *p*-value < 0.0001) (Figure 1A). The alkaloid extract was tested as well, but no significant difference was observed between AlkE treated and untreated worms (Figure 1B).

To evaluate the influence of GE on the basal level of ROS accumulation in vivo, N2 (wild type) worms were exposed to the molecular probe H2DCFDA; the probe is sensitive to oxidation by H_2_O_2_ produced during the cellular aerobic metabolism. Once oxidized, it converts into DCF, which is a highly fluorescent compound. Analyses of the emitted florescence indicated a significant difference between GE treated and untreated worms; a decrease of up to 30% was observed in worms treated with 300 μg/mL GE compared to the untreated control group (adjusted *p*-value < 0.0001) (Figure 1C). The result adds up to the demonstrated capacity of GE to protect worms against juglone induced oxidative stress, supporting the role of GE as antioxidant in vivo. Interestingly, the AlkE extract that was inactive in the survival assay was able to decrease ROS accumulation by about 30% in worms treated with 300 μg/mL compared to the untreated control group (adjusted *p*-value < 0.0001) (Figure 1D).

**Figure 2:**
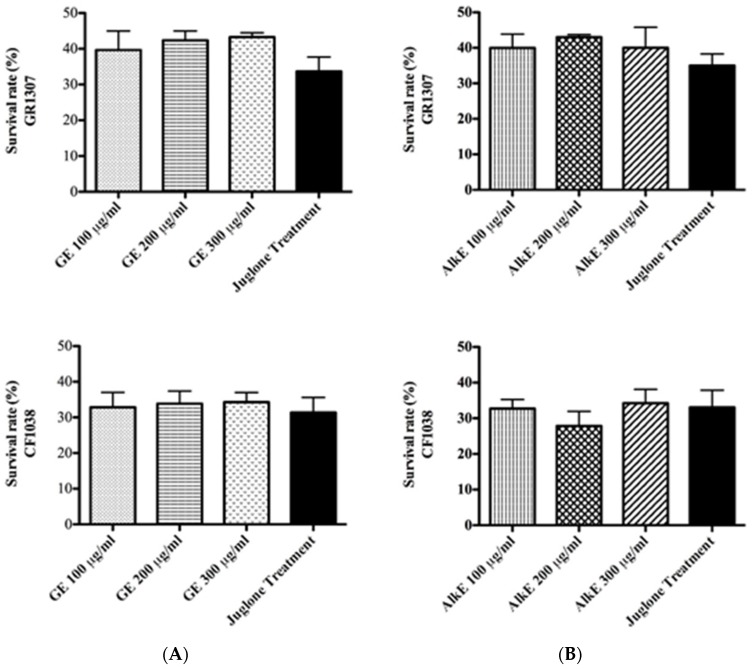
Effect of GE (**A**) or AlkE (**B**) treatment on the survival rate of the mutant strains CF1038 and GR1307 under juglone-induced oxidative stress. The DAF-16 mutant worms (GR1307 [daf-16(mgDf50) I] and CF1038 [daf-16(mu86) I]) did not respond to GE or AlkE treatment. Each bar represents the mean ± SEM from three independent experiments. The treatment groups are compared with the untreated control by one-way ANOVA followed by Bonferroni’s method (post-hoc).

To assess whether the antioxidant effect of GE is based on its capacity to scavenge radicals or if it involves a molecular mechanism of stress resistance, we carried out the survival assay using mutant worms which lack the *daf-16* gene (CF1038 ([daf-16(mu86)I]), GR1307 ([daf-16(mgDf50)])). By this approach, the inability of the extract to counteract the toxic effect of juglone could be demonstrated (Figure 2A), in contrast to the result with N2 (wt) worms. The alkaloid extract was tested as well; but, no significant difference was observed between AlkE treated and untreated worms (Figure 2B). Therefore, we assume that the antioxidant activity of GE is an outcome of its capacity to modulate molecular pathways of stress resistance, particularly involving the DAF-16 transcription factor.

DAF-16 is the *C. elegans* homologue of the mammalian FOXO transcription factor, which regulates the expression of several genes involved in stress resistance and longevity. Furthermore, mammalian FOXO proteins have been associated with aging phenotypes and aging related diseases, such as Alzheimer’s disease, cancer and diabetes type II [25].

### 3.4. Quantification of the Expression of Stress Response Genes after Guarana Treatment

A suitable approach to evaluate the efficacy of antioxidants in vivo is to assess the expression pattern of stress response genes like *hsp*-16.2 and *sod*-3; in this context, *C. elegans* is an appropriate model organism as several relevant mutant strains with a GFP reporter gene fusion are available.

**Figure 3:**
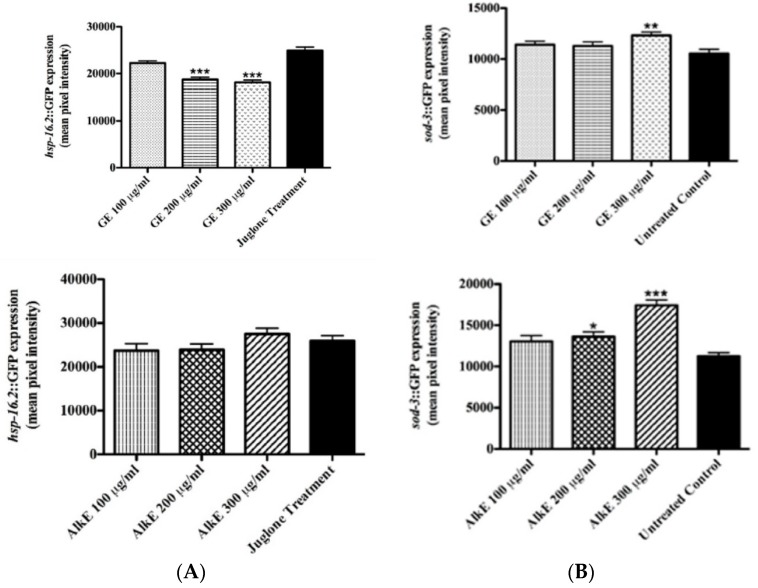
Effect of GE or AlkE treatment on the expression of stress resistance related genes. (**A**) Heat shock protein 16.2 (HSP-16.2) expression in the mutant strain TJ375 [hsp-16.2::GFP(gplsI)] under juglone-induced oxidative stress. The *hsp*-16.2 expression levels were significantly decreased by GE treatment compared to the untreated control. No significant effect was observed among worms treated with the alkaloid extract, AlkE. (**B**) Superoxide dismutase 3 (SOD-3) expression in the mutant strain CF1553 after treatment with GE and AlkE. Worms [(pAD76)sod-3p::GFP + rol-6] treated with 300 μg/mL of GE showed higher levels of SOD-3 expression compared to the control. Similar results were observed among worms treated with the alkaloid extract, AlkE. The results are presented as mean ± SEM from three independent experiments. * *p* <0.05, ** *p* <0.01, *** *p* < 0.0001 compared to the untreated control by one-way ANOVA followed by Bonferroni’s method (post-hoc).

To assess the influence of GE on *hsp*-16.2 expression, TJ375 worms expressing the target gene fused with a GFP reporter, pre-treated with GE or AlkE, were submitted to mild oxidative stress induced by 20 μM juglone. Analyses of the emitted fluorescence revealed a significant difference among the groups: GE treated worms exhibited up to 23% lower fluorescence intensity compared to the untreated control worms (adjusted *p*-value < 0.0001) (Figure 3A). No significant effect was observed among worms treated with AlkE extract (Figure 3B). *hsp*-16.2 expression is frequently referred as a stress sensor due to the fact that it takes place only when the organism is facing stress conditions, such as heating or oxidants [26].

The expression of *sod-3*, a gene that codes for the mitochondrial antioxidant enzyme superoxide dismutase 3, was investigated using the mutant strain CF1553 (muIs84 ([(pAD76)sod-3p::GFP+rol-6]). The results showed significantly higher fluorescence intensity among mutants treated with 300 μg/mL GE as compared to the untreated control group (adjusted *p*-value = 0.0033) (Figure 3C). Similar results were obtained from worms treated with the alkaloid extract (Figure 3D). At the highest tested concentration of 300 μg/mL AlkE, the GFP expression was up 40% higher in comparison with the untreated control (adjusted *p*-value < 0.0001).

The demonstrated ability of GE to counteract juglone toxicity (down-regulate *hsp*-16.2) and to up-regulate *sod*-3 reinforces the assumption that the extract effectively promotes antioxidant activity in vivo. AlkE treatment also promoted an increase in the expression of *sod-3*::GFP, but did not influence the expression pattern of *hsp*-16.2::GFP. Although similar, the effect promoted by GE seems more interesting with regard of the antioxidant activity than the alkaloid extract.

### 3.5. Anti-Aging effects

**Figure 4:**
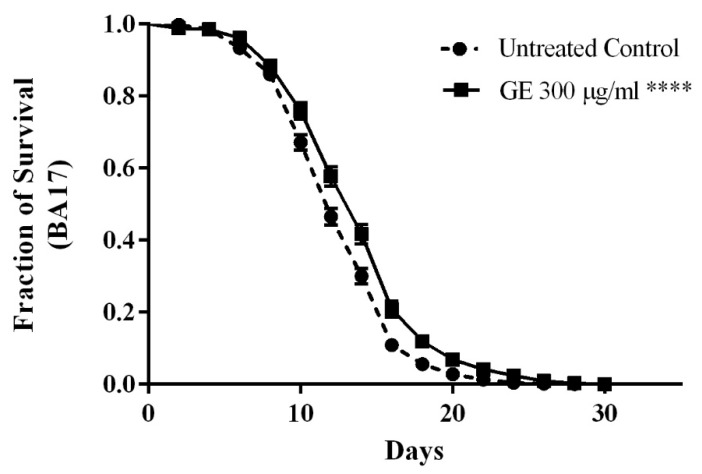
Influence of GE treatment on longevity of the mutant worms BA17. GE 300 μg/mL increased the lifespan significantly as compared to the control group. The results are presented as percentage of survival and the statistical significance was determined by Log-rank (Mantel-Cox) tests followed by Gehan-Breslow-Wilcoxon Test. **** *p* < 0.0001.

The putative ability of guarana to extend the lifespan was explored in *C. elegans* similarly to other polyphenol rich extracts like green tea and rooibos tea [18,27]. For the assay, feminized mutants BA17 (fem-1(hc17), which are unfertile when cultured at 25 °C, were selected. A significant extension of the lifespan by 14% was scored in worms treated with 300 μg/mL GE as compared to untreated control worms (*p*-value *p* < 0.0001) (Figure 4).

**Figure 5:**
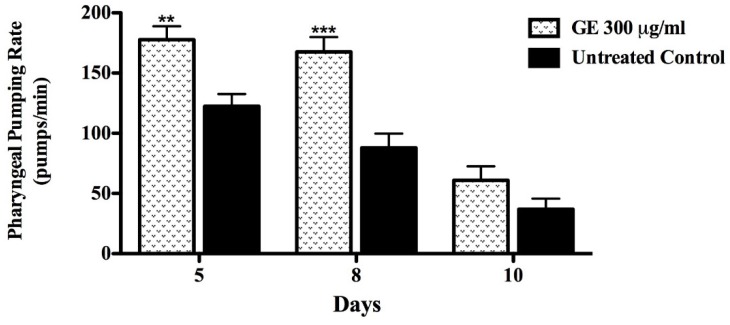
Pharyngeal pumping rate in N2 wt worms after GE treatment. The treatment with GE 300 μg/mL significantly attenuated the age-associated decline in the muscle function of pharynx. Data are presented as mean ± SEM. ** *p* <0.01,*** *p* < 0.0001 related to the control by a two-way ANOVA.

Caloric restriction (CR) is known as a potent lifespan enhancer [28]. In order to investigate whether worms treated with GE underwent caloric restriction during the course of the experiment, the pharyngeal pump rate was measured (which would decrease in case of CR). The pumping activity was scored in GE treated and untreated N2 (wt) worms at day 5, 8 and 10 of adulthood. The results obtained indicate a positive effect of GE throughout all periods of observation. The pumping activity of the GE treated group was 60% higher at day 10 compared to the untreated control group (*p*-value < *p* < 0.0001) (Figure 5). 

No impairment of the pumping activity of the pharynx was observed among GE treated worms; instead, the pumping rate was significantly higher compared to that scored among the untreated control. We assume that GE lifespan extension effect is not due to CR influence. Moreover, the attenuation of the age-related muscle function decline, assessed in *C. elegans* by the activity of the pharynx, is indicative for the anti-aging property of guarana extracts [29,30]. *C. elegans* pharynx muscle structure undergoes progressive deterioration and function decline with age, similarly to the sarcopenia which accompanies human aging; the damage seems to be at least partially the result of a lifetime stress promoted by increased ROS generation due to the high ATP demand of those muscles [31].

**Figure 6:**
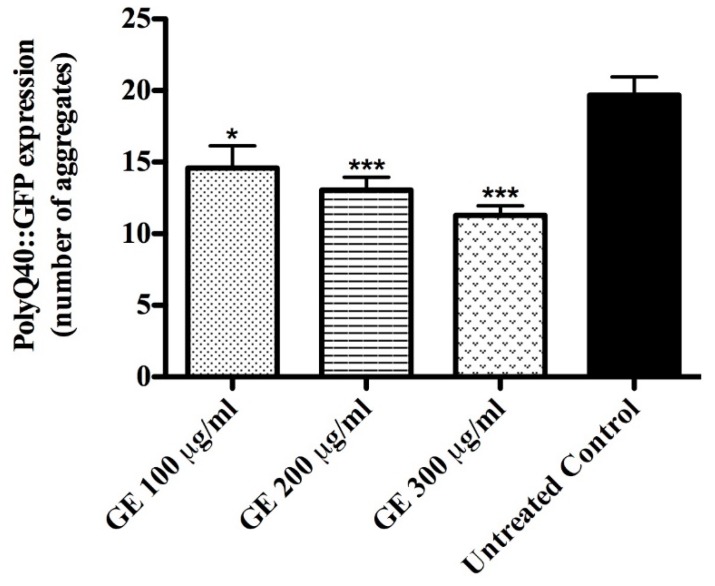
PolyQ40::YFP aggregate accumulation in mutant worms AM141 after GE treatment**.** Worms treated with GE exhibited significantly lower numbers of polyQ40::GFP aggregates compared to the control group. Data are presented as mean ± SEM. * *p* <0.05, ** *p* < 0.01 and *** *p* < 0.0001 related to the control by a one-way ANOVA followed by Bonferroni (post-hoc).

Genetic studies with *C. elegans* showed a positive association between aging delay and neuroprotection[32]; correspondingly, the number of polyQ40 aggregates, scored using the mutant strain AM141 ([rmIs133 (unc-54p::Q40::YFP)]), was significantly lower among GE treated worms compared to the untreated control group. When tested at a concentration of 300 μg/mL GE, the number of aggregates was 30% lower than in the corresponding control group (adjusted *p*-value < 0.0001) (Figure 6); the data supports the anti-aging property of guarana.

**Figure 7:**
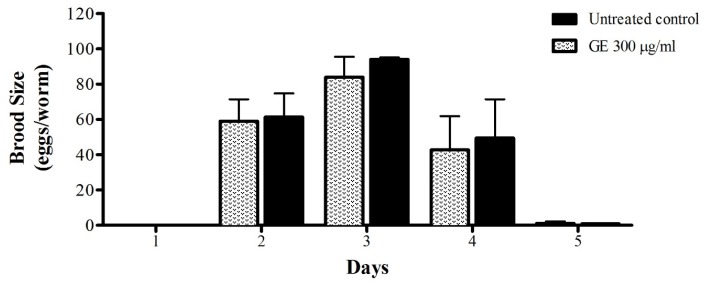
Brood size of N2 (wt) worms after GE treatment. Treatment with GE 300 μg/mL had no effect on egg laying activity. The results are expressed as mean ± SEM from three independent experiments. Treatment groups are compared to the untreated control by two-way ANOVA.

Bridi et al. [33] demonstrated the lifespan-prolonging effect of caffeine in *C. elegans*. However, the mentioned effect was accompanied by delayed larval development and impaired fertility rate. Sutphin et al. [34] reported the ability of caffeine to attenuate age-associated markers, including formation of polyQ40 plaques and highlighted that the ability of caffeine to extend the lifespan is temperature-dependent; the substance significantly shortened the lifespan of wild-type worms cultured at 25 °C. Guarana extract has a high caffeine content and also exhibited a lifespan prolonging effect and attenuation of aging markers in our study; however, no significant impairment in the fertility rate during the entire fertile period was observed (Figure 7).

Caffeine might contribute to the antioxidant and longevity-promoting properties of guarana but it is not expected to be the sole active compound in that extract. The purine alkaloids may play a synergistic role in the antioxidant activity of guarana together with the known antioxidant phenolic components, including tannins (catechin, epicatechin and their polymers) and proanthocyanidins.

## 4. Conclusions

In summary, the current work demonstrated substantial antioxidant and anti-aging activity of guarana in *C. elegans* and indicates a new utilization for the fruit that is already well known due to its stimulant effects. The water extract from guarana can apparently enhance stress resistance though a pathway, which is dependent on the activity of the transcription factor DAF-16/FOXO. In addition, the extract can extend the lifespan and attenuate markers of aging, such as the age-related muscle function decline, assessed through the pharynx activity, and the formation of polyQ40 aggregates. However, further studies are needed to understand the underlying mechanisms and to identify the possible health benefits of guarana consumption throughout the human aging process.

## Figures and Tables

**Table 1:** Determination of guarana extract (GE) chemical content. The high concentration of caffeine is a hallmark of this species.

Compound	Concentration (mg/g)
Caffeine	102.8
Theophyline	2.3
Thebromine	1.0
Catechin	+
Epicatechin	+

+ Detected by ESI-MS/MS analysis but not quantified.

**Table 2:** In vitro antioxidant activity of GE as compared to the alkaloid extract from guarana (ALK) and standard natural antioxidants.

Sample	DPPH *
GE	40
EGCG	1.03
Vitamin C	2.12
ALK	-

* EC_50_ values expressed in μg/mL sample; - no antioxidant activity detected.
